# Sustainable Chromium Recovery From Wastewater Using Mango and Jackfruit Seed Kernel Bio-Adsorbents

**DOI:** 10.3389/fmicb.2021.717848

**Published:** 2021-09-28

**Authors:** Deen Dayal Giri, Maulin Shah, Neha Srivastava, Abeer Hashem, Elsayed Fathi Abd_Allah, Dan Bahadur Pal

**Affiliations:** ^1^Department of Botany, Maharaj Singh College, Saharanpur, India; ^2^Environmental Technology Limited, Ankeleshwar, India; ^3^Department of Chemical Engineering, Indian Institute of Technology (BHU) Varanasi, Varanasi, India; ^4^Botany and Microbiology Department, College of Science, King Saud University, Riyadh, Saudi Arabia; ^5^Plant Production Department, College of Food and Agricultural Sciences, King Saud University, Riyadh, Saudi Arabia; ^6^Department of Chemical Engineering, Birla Institute of Technology, Mesra, Ranchi, India

**Keywords:** biosorption, chromium, isotherm, jackfruit, kinetic, mango, seed, kernel

## Abstract

Wastewater is a rich source of valuable chemicals of industrial importance. However, their economic recovery is crucial for sustainability. The objective of the present work is to recover hexavalent chromium (Cr VI) as a value-added transition metal from wastewater cost-effectively; the biosorbent derived from seed kernels of mango (M) and jackfruit (JF) were applied for removing the metal from simulated wastewater. The functional groups of the biomass were analysed with the help of Fourier transform infrared (FTIR) spectroscopy, micrographs were generated using a scanning electron microscope, and crystallinity was determined by an x-ray diffractometer (XRD). The concentration of Cr VI in wastewater was analysed by an inductively coupled plasma optical emission spectrometer (ICP-OES). Process parameters (pH, dose, contact time, temperature, and initial concentration) were optimized for efficient Cr VI adsorption using a response surface methodology-based Box–Behnken design (BBD) employing Design-software 6.0.8. The batch experiment at room temperature at pH 4.8 and Cr VI removal ∼94% (M) and ∼92% (JF) was achieved by using a 60-mg dose and an initial Cr (VI) concentration of 2 ppm in 120 min. The equilibrium Cr binding on the biosorbent was well explained using Freundlich isotherm (*R*^2^ = 0.97), which indicated the indirect interactions between Cr (VI) and the biosorbent. Biosorption of Cr (VI) followed the pseudo-order and intra-particle diffusion models. The maximum adsorption capacity of the M and JF bio-adsorbent is 517.24 and 207.6 g/mg, respectively. These efficient, cost-effective, and eco-friendly biosorbents could be potentially applied for removing toxic Cr (VI) from polluted water.

## Introduction

In the recent past, rapid industrialization and over-exploitation of resources have drastically polluted our natural water bodies with many toxic heavy metals. The presence of heavy metals severely degraded the quality of the aquatic habitat. Wastewater generated from various industries like leather, paper, pulp, pigment, and electroplating carries significant amount of toxic metal (Behera et al., 2020; Dhiman et al., 2020). The harmful effect of heavy metal varies from metal to metal; however, their trace concentration in parts per million (ppm) or parts per billion (ppb) could be much harmful in chronic exposure to humans and animals (Bharath et al., 2020). After entry into the environment, the non-biodegradable toxic heavy metals persist and enter the food chain and adversely affect different living organisms. So, it is necessary to restrict the discharge of heavy metals in the aquatic ecosystem and remediation of polluted waterbody to ensure good health for animals including humans.

Chromium occurs in the earth’s crust in many oxidation states of Cr (II–VI). However, the stable and prevalent oxidation states are trivalent (Cr III) and (Cr VI), which exist in aquatic and terrestrial ecosystems (Shahid et al., 2017; Sawalha et al., 2020). Trace amount of chromium is required in mammalian metabolism of protein, fat, and sugar, but no such requirement has been reported in plants or microorganisms. The high concentration of Cr is always toxic to animals, and its toxicity varies depending on the oxidation state. The hexavalent form (Cr VI) of chromium is more toxic compared to the trivalent form (Cr III). The ill effects of Cr VI include diarrhoea, ulcer, irritation of eye and skin, dysfunction of kidney, and carcinoma of the lung (Costa, 2003; Mohanty et al., 2005). It adversely affects reproductive health and causes birth defects (Kanojia et al., 1998). High dose results in the death of the animal and human, with an LD50 of approximately 50–100 mg in oral intake for rats (De Flora et al., 1990). Chromium affects plant growth by inhibiting photosynthesis (Shahid et al., 2017). Plant cells exposed to Cr (VI) exhibit oxidative stress and chromosome damage by forming adduct with the DNA. Such interaction of Cr (VI) with genetic material causes genome instability and epigenetic alterations leading to carcinogenesis in animals and humans (Pavesi and Moreira, 2020). The entry of Cr (VI) in the metabolism is mainly through water consumption. The safe limit of Cr in drinking water as per the Environmental Protection Agency (United States) is less than 0.1 ppm (Rambabu et al., 2019). Despite the higher concentration limit, Cr has industrial applications in the area of utensils and stainless steel pots, and as a pigment and chemical (Gaudiuso et al., 2010). Approximately 80% of Cr has been mixed in stainless steel utensils.

Chromium VI can be eliminated from polluted water using different techniques. Some of the important procedures used are adsorption (Rangabhashiyam et al., 2016), coagulation, precipitation, membrane separation (Rambabu and Velu, 2016), ion-exchange method, and electrochemical technique (Rangabhashiyam et al., 2016). The issue of Cr and other metal pollutants can also be overcome by the use of novel photocatalysts from biopolymers, biochar, carbons, enzymes, and proteins (Kumar et al., 2020). The photocatalysts can rapidly reduce Cr VI into Cr III by the use of a nano-composite of ZnO-Fe_2_O_3_ (Dhiman et al., 2020). Despite the availability of many options, cost-effective sustainable green processes are always preferred. The use of biochar of various origin is an example of such processes that has attracted attention for remediating chromium-polluted aquatic systems (Lee et al., 2019). The adsorption process is adapted mainly due to its efficiency, selectivity, low operational cost, and reusability. This is a low-cost green method that utilizes naturally occurring materials for adsorbing toxic metal ions from water (Noormohamadi et al., 2019). The plant materials vary in the chemical composition depending on plant species, plant part, adaptation, and habitat (Saygılı et al., 2018). The chemical composition and type of phytochemical present in the biosorbent is likely to affect the biosorption of metal from the water. Biosorbents from different ecological adaptation that have been tested for their metal biosorption potential are aquatic macroalgae *Enteromorpha* (Rangabhashiyam et al., 2016), aquatic plant *Vallisneria gigantea* (Iriel et al., 2015), husk of high-water requiring paddy (Varala et al., 2019), terrestrial tree *Pongamia pinnata* seed (Brungesh et al., 2015), leaves of *Colocasia esculenta* (Nakkeeran et al., 2016) and *Ficus auriculata* (Rangabhashiyam et al., 2015), and bagasse of sugarcane (Ullah et al., 2013). The main issue with the testing of materials is their low adsorption capacity (Nirmala et al., 2019). Nuts of the medicinal plant *Terminalia arjuna* activated with zinc chloride have been used for removing chromium from water (Mohanty et al., 2005). Recently, we prepared the biosorbent from seeds of Java Plum and Amaltash that efficiently removed arsenic from the synthetic wastewater below the safe limit (Giri et al., 2021). In the present investigation, biosorbent was prepared from the kernel of two tropical trees, jackfruit (*Artocarpus heterophyllus*) and mango (*Mangifera indica*). The trees are abundantly present across India and large numbers of seed biomass are easily available for preparing biosorbent.

Thus, the objective of the present study was the recovery of Cr from the synthetic wastewater using biosorbent prepared from the kernel of jackfruit and mango in the batch experiment. In the optimized operating experimental conditions, the selected biosorbents were successfully applied in Cr VI removal from synthetic wastewater.

## Materials and Methods

### Investigational Setup and Procedure

The investigational arrangement was used to execute in batches the adsorption procedure as described earlier (Pal et al., 2017; Giri et al., 2021). In brief, the experiment was conducted in a 50-ml beaker having a magnetic stirrer (up to 500 rpm and least count 5 rpm). The chromium concentration in the beaker varied (0.8–2.5 ppm) for the varying bio-adsorbent dose (20–80 mg). The experiments were conducted for 120 min for optimizing the bio-adsorbent dose, pH, and initial Cr VI concentration of the aqueous solution. Samples were taken out at an interval of every 10 min for determining the remaining metal concentration in the solution.

### Bio-Adsorbent Preparation

The biomasses were collected locally in BIT Mesra Ranchi, Jharkhand, India campus. The surface washing of samples was done under running tap water (10 min) to remove surface-adhered soil particles. The washed seeds were oven-dried (48 h, 60°C), crushed, milled, and passed through a mesh to obtain uniform-sized biomass in powder form. The powdered biomass was used in characterization and calcination (400°C, 3 h).

### Batch Adsorption Experiments

The heavy metal adsorption was studied at a specific initial Cr VI concentration and definite dose of bio-adsorbent and at a fixed pH of a solution. Separate experiments were performed at initial Cr VI concentrations of 0.8, 1.0, 1.5, and 2.5 ppm for each dose of 20, 40, 60, and 80 mg. Furthermore, the pH of the solution was optimized at a specific initial concentration and dose by experimenting with different pH (3, 4.8, 8, and 10) under constant stirring conditions at room temperature for 120 min. Samples were withdrawn at the specific interval and analysed for Cr VI concentration using ICP-OES. The data obtained were applied in kinetics analysis and equilibrium tests were performed.

### Adsorption Isotherm

For the adsorption study, three classical models devised by Langmuir, Freundlich, and Temkin were used in the present investigation, which is represented below. The Langmuir model is presented well by the equation given below (Langmuir, 1916, 1918):


(1) $$\frac{{\text{C}}_{\text{e}}}{{\text{q}}_{\text{e}}}=\frac{1}{{\text{q}}_{\text{m}}\,{\text{b}}_{0}}+\frac{{\text{tC}}_{\text{e}}}{{\text{q}}_{\text{m}}}$$

The Freundlich model linear form of the model equation (Freundlich, 1906; Foo and Hameed, 2010) is shown below:


(2) $$\ln{\text{q}}_{\text{e}}=\ln{\text{K}}_{\text{f}}+\frac{1}{\text{n}}\,\ln{\text{C}}_{\text{e}}$$

The general linear equation of the Temkin model (Temkin and Pyzhev, 1940) is presented as follows:


(3) $${\text{q}}_{\text{e}}=\frac{\text{RT}}{{\text{b}}_{\text{T}}}\,\ln{\text{K}}_{\text{T}}+\frac{\text{RT}}{{\text{b}}_{\text{T}}}\,\ln{\text{C}}_{\text{e}}$$

### Adsorption Kinetics Models Analysis

The sorption process depends on the physiochemical features of the adsorbent as well as the conditions of the system (Nadeem et al., 2006). The number of metals adsorbed is determined by using the equation:


(4) $${\text{q}}_{\text{Cr}}=\frac{\left({\text{C}}_{0}-{\text{C}}_{\text{e}}\right)\,\text{V}}{\text{W}}$$

The pseudo-first-order model is expressed as follows (Farhan et al., 2012):


(5) $$\log\left({\text{q}}_{\text{e}}-{\text{q}}_{\text{t}}\right)=\log{\text{q}}_{\text{e}}+\frac{{\text{k}}_{1}}{2.303}\,\text{t}$$

The linear form of the pseudo-second order rate expression is given below:


(6) $$\frac{\text{t}}{{\text{q}}_{\text{t}}}=\frac{1}{{\text{q}}_{\text{e}}^{2}\,{\text{k}}_{2}}+\frac{\text{t}}{{\text{q}}_{\text{e}}}$$

The diffusion kinetic can be written as (Furusawa and Smith, 1974):


(7) $${\text{q}}_{\text{t}}={\text{t}}^{0.5}\,{\text{k}}_{\text{ipd}}+\text{C}$$

The linear form of the Elovich model can be written as (Zhang et al., 2019):


(8) $${\text{q}}_{\text{t}}=\frac{1}{\beta }\,\log\left(\alpha \,\beta \right)+\frac{1}{\beta }\,\log\text{t}$$

### Characterization

A morphological study of the bio-adsorbent surface was done by using a field emission scanning electron microscope assisted with EDX (Sigma-300 with EDX, Ametek), and IR-Prestige 21 was used for recording the Fourier transform infrared (FTIR) spectrum of the catalyst in the range of 400–4000 cm^–1^ (Shimadzu Corporation, Japan). For recording x-ray diffraction patterns, a diffractometer with a Cu-Kα radiation at 40 kV and 40 mA was used (Rigaku, Smart Lab 9 kW diffractometer, Japan). The analysis of Cr VI in the experiment was done using an inductively coupled plasma optical emission spectrometer (ICP-OES) from Perkin Elmer (United States), with the ability to analyse 20–25 elements within 5 min, Optical 2100DV in the spectral range 160–900 nm, and a resolution of 0.009 nm at 200 nm.

### Experimental Design

Chromium removal from wastewater by the use of biowaste is a newly developed technology. This technology uses easily available waste material to treat chromium-containing wastewater. The experiments have been performed by using different concentration levels of biowaste adsorbents. Two types of adsorbents have been used to perform the experiments as discussed earlier by using Design-Expert 6.0.8 software to design experiments. Process parameters like pH, adsorbent doses, and chromium concentration have been optimized based on % removal. Equilibrium investigation has been performed by using Design-Expert 6.0.8 software. For the response surface method, the Box–Behnken design (BBD) has been chosen in this study. A three-level and three-factor design was applied and the range and levels are shown in Table 1. The full factorial design shows a total number of 17 experiments by using each adsorbent. All the results obtained in the form of % removal have been fed to the DOE for developing a second-order polynomial model. A quadratic response model was developed using all the linear, quadratic, and interaction terms as described in the equation given below:


(9) $$Y=C_{0}+\sum C_{i}\,X_{i}+\sum C_{i\,i}\,X_{i\,i}^{2}+\sum C_{i\,j}\,X_{i}\,X_{j}+\in$$

**TABLE 1:** Experimental range and levels of the independent test variables.

**Variables**	**Unit**	**Range and level**		
		**Low**	**Medium**	**High**
pH	–	2	5	8
Adsorbent doses	*ppm*	40	60	80
Concentration	*ppm*	1	1.5	2

In the equation (9), predicted yield is represented by *Y*, which depends on *C*_0_ (constant), *C*_*i*_ (linear coefficients), *C*_*ii*_ (quadratic coefficients), and *C*_*ij*_ (cross-product coefficients).

## Results and Discussion

### FE-SEM and ICP-OES Analysis

**FIGURE 1:**
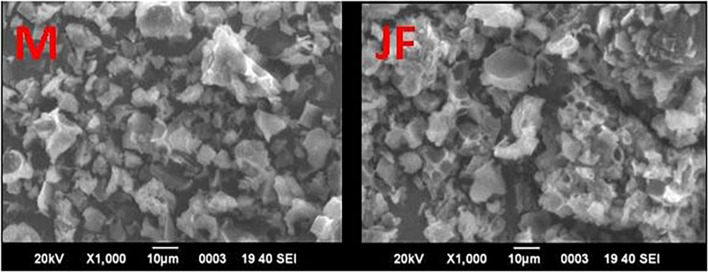
SEM micrograph of bio-sorbent material of Mango (MF) and Jack fruit (JF) kernel biomass.

The FE SEM micrographs of the mango seed kernel (M) and jackfruit seed kernel (JF) power calcined (400°C, 3 h) are given in Figure 1. A non-uniform irregular flake structure has been observed for both the biomass seed. Based on prior calcination biomass powder analysis by ICP-OES, the major metal composition recorded was iron, calcium, magnesium zinc, manganese, nickel, copper, and cobalt. Earlier investigations also showed the presence of such elements. Theivasanthi et al. (2011) showed the presence of C, O, Mg, Al, P, K, Ti, Fe, Ni, and Mo in the biomass samples.

### Fourier Transform Infrared Analysis (FTIR)

**FIGURE 2:**
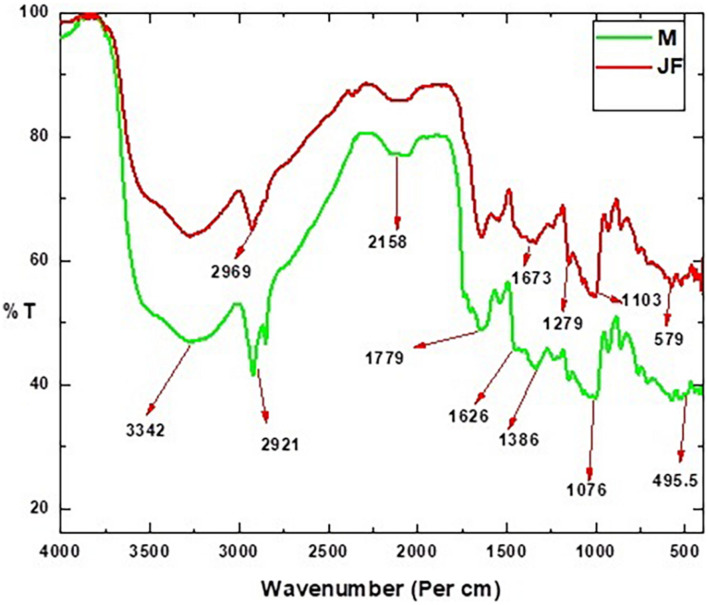
FTIR spectra of bio-sorbent material of Mango (MF) and Jack fruit (JF) kernel biomass.

The different useful groups existing on the biosorbent surface in the FTIR spectra are depicted in Figure 2. The peculiar peaks were observed at wave number 3,289, 2,921, 2,158, 1,626, 1,485, 1,349, 1,076, and 595 cm^–1^ in the FTIR spectra. The stretch observed in the range of 3,289–3,272 were due to involvement of –OH and NH_2_ in Cr VI binding (Krishnamoorthy et al., 2019). The position of the peak at 2,921 is due to the C–H stretching in the seed biosorbents. The peaks in the range of 2,927–2,890 were assigned to C–H bond stretching (Rangabhashiyam et al., 2015) and in the seed biosorbent of *Moringa oleifera* (Araujo et al., 2010). The peak detected at 1,626 cm^–1^ and 1,485 cm^–1^ could be attributed to –C=C- and –C=O (Kumar et al., 2019). The C=C stretch has been shown at 1,588 by Rambabu et al. (2020). The peak near 1636 has been assigned to the –COOH group and that near 1020 has been assigned to the -CN group (Liu et al., 2013). The peak at 1076 could be due to the stretching vibration C–O bond of alcohol and carboxylic acid as assigned by Rambabu and co-workers. The peak at 1643, 1419, and 1060 were attributed to the stretching vibration of amide, C-H, and C-O bond of alcohols in the powdered seed biomass of *Strychnos potatorum* by Jayaram and Prasad (2009). The carboxyl group present in the bio-adsorbent plays an important role in adsorbing the heavy metal cations (Kim et al., 2015). The existence of glacial groups on the biomass-based carbon plane could help in the significant cation substitution of the absorbent (Abdel-Ghani et al., 2009).

The peak near 595 cm^–1^ could be assigned to Fe-O (Chen et al., 2011). The cation exchange on the bio-adsorbent surface is enhanced by polar groups such as C=O, which helps in their adsorption (Abdel-Ghani et al., 2009; Kim et al., 2015).

### X-Ray Diffraction (XRD)

**FIGURE 3:**
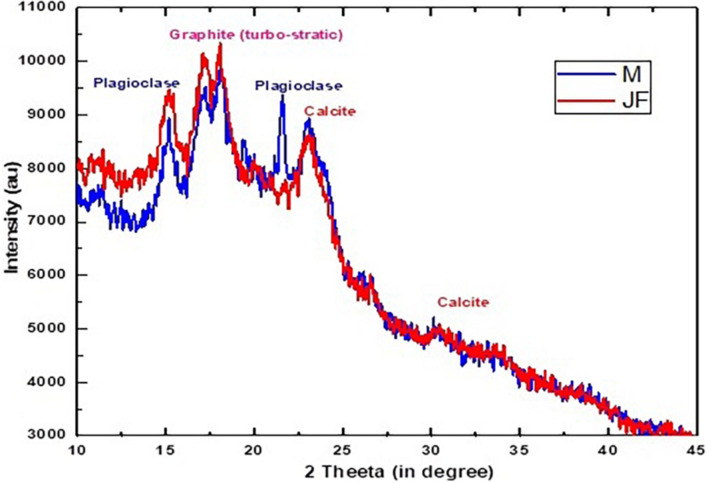
XRD spectra of bio-sorbent material of Mango (MF) and Jack fruit (JF) kernel biomass.

The XRD analysis of mango (M) and jackfruit (JF) kernel biomass is shown in Figure 3. Mostly, the amorphous phase was present in the MF and JF calcined at 400°C for 3 h and the crystalline part was very small. A number of Bragg reflections can be seen, which correspond to 2θ value at 15.12, 17.57, 23.01, 31.18, and 38.23 and the corresponding (h, k, l) values are (1, 1, 1), (2, 0, 0), (2, 1, 1), (3, 1, 1), and (3, 2, 2), respectively (Theivasanthi et al., 2011).

### Kinetics Study and Equilibrium Isotherm

**FIGURE 4:**
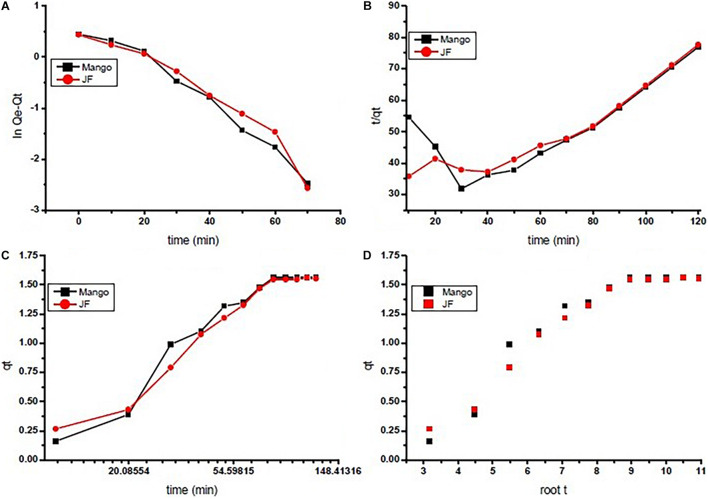
Adsorption kinetics of chromium by using seed bio-sorbent of Mango (M) and Jackfruit (JF): **(A)** first order, **(B)** second order, **(C)** Elovich, and **(D)** interparticle.

The reaction kinetics used in the present investigation for chromium adsorption using mango (M) and jackfruit (JF) kernel biomass-based bio-adsorbent is shown in Figure 4. The data show the fast initial Cr adsorption with gradual slowdown with time after 30–40 min of approximately 70%, which further increased to attain the final adsorption level of 94% (M) and 92% (JF) at the 60-mg dose, pH 4.8, and an initial concentration of 2 ppm. While using both the adsorbents (M and JF), steady state was attained in 80 min; however, the experiment was performed for 120 min at room temperature keeping pH at 4.8 to ensure equilibrium adsorption. In the present study, four different kinetic models were tested to evaluate the kinetics of the Cr VI adsorption on the biosorbents. Such type of model testing has been reported earlier (Furusawa and Smith, 1974; Farhan et al., 2012). These models are the pseudo-first-order, pseudo-second-order, intra-particle diffusion, and Elovich models, which are represented in the form of linear equation in Eqs 5–8, respectively.

In Table 2, these pseudo-first-order, pseudo-second-order, intra-particle, and Elovich models describe the experimental data well with high coefficients. The biosorbent followed the Freundlich adsorption model based on the regression coefficient for linear fitted data (*R*^2^ = 0.95 M, *R*^2^ = 0.96 JF). The kinetics of Cr VI adsorption was explained in terms of pseudo-first order (*R*^2^ = 0.97 M, *R*^2^ = 0.97 JF). The maximum adsorption capacity of M and JF bio-adsorbent is 517.24 and 207.6 g/mg, respectively, whereas the equilibrium adsorption capacity was 5.21 and 2.23 g/mg, respectively. Comparison of the kinetic constraints of various kinetic models and their regression constant is tabulated in Table 3, which suggests better values in the present study compared to others. These similar adsorbent results also showed examples of nickel-zinc ferrite (Ahangari et al., 2019), bark (Aregawi and Mengistie, 2013), starch maghemite (Siddiqui et al., 2020), and seed (Aregawi and Mengistie, 2013; Prasad et al., 2020; Pal et al., 2021).

**TABLE 2:** Comparative kinetic parameters of Cr (VI) adsorption onto seeds bio-sorbent of Mango (M) and Jack fruit (JF) kernel biomass.

**Kinetic model**	**Parameters**	**M (Present study)**	**JF (Present study)**	**Starch maghemite (Siddiqui et al., 2020)**	**Watermelon waste (Khattak et al., 2017)**	**HCl to develop modified roots labeled (AHP) (Shooto, 2020)**	**Harpagophytum procumbens plant (PHP) (Shooto, 2020)**
Pseudo-first order	k_1_ (min^–1^)	0.095	0.055	0.06	0.01589	0.0583	0.0551
	q_*e*_ (mg/g)	5.21	2.23	2.76	32894	103.1	76.81
	*R* ^2^	0.97	0.97	0.60	0.9745	0.96	0.99
Pseudo-second order	k_2_ (g/mg. min)	0.00545	0.0094	9.16	2.32 × 10^–6^	0.0137	0.0185
	q_*e*_ (mg/g)	2.65	2.26	1.03	32894	128.9	97.24
	*R* ^2^	0.53	0.79	0.99	0.992	0.88	0.972
Intra-particle model	K_*ipd*_ (mgg^–1^ min^–1/2^)	0.171	0.182	0.03	NA	12.26	9.073
	C (mg g^–1^)	0.091	0.157	0.62	NA	39.70	23.71
	*R* ^2^	0.91	0.92	0.90	NA	0.97	0.98
Elovich model	β (g mg^–1^)	2.496	2.59	9.93	NA	NA	NA
	α (mgg min^–1^)	2.894	2.59	9.76	NA	NA	NA
	*R* ^2^	0.94	0.95	0.81	NA	NA	NA

**TABLE 3:** Summary of equilibrium parameters of Cr (VI) adsorption on Mango (M) and Jack fruit (JF) kernel biomass.

**Isotherm model**	**Parameters**	**M (Present study)**	**JF (Present study)**	**Activated carbon (Yao et al., 2014)**	**Farmyard manure bio-char (Idrees et al., 2018)**	**Poultry manure based bio-char (Idrees et al., 2018)**	**Seed (Aregawi and Mengistie, 2013)**
Langmuir model	b_*o*_ (L/mg)	0.017	0.013	0.1816	0.092	1.056	0.82
	q_*m*_ (mg/g)	517.24	207.6	17.86	6.652	2.403	3.23
	*R* ^2^	0.73	0.74	0.9998	0.998	0.479	0.999
Freundlich model	K_*f*_ (mg/g)	2.68	2.46	7.3161	0.520	1.103	1.02
	1/n	0.667	0.578	0.1808	0.765	0.536	0.086
	*R* ^2^	0.95	0.96	0.9806	0.984	0.809	0.966
Temkin model	b_*T*_ (J mol^–1^)	970.50	1103.63	NA	2942	3603	31568
	K_*T*_ (L g^–1^)	9.29	26.34	NA	9.634	285.6	2.29
	*R* ^2^	0.89	0.92	NA	0.922	0.949	0.971

**FIGURE 5:**
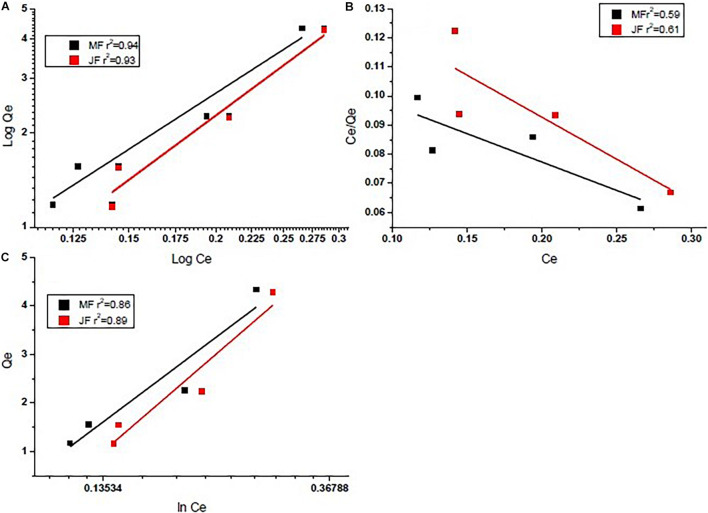
Equilibrium isotherms of chromium by using seed bio-sorbent of Mango (M) and Jackfruit (JF): **(A)** Freundlich model, **(B)** Langmuir model, and **(C)** Tempkin model.

Investigational information was analysed for deciding the finest adsorption isotherm describing the adsorption process on the selected bio-adsorbents. The adsorption mechanism in Eqs 1–3 for three different models such as Langmuir, Freundlich, and Tempkin isotherms were tested for data fitting. The best-suited isotherm was selected based on the value of *R*^2^ for linear fit (Kan et al., 2017). The outcome of isotherm studies is presented in Figures 5A–C and is also shown in Table 3. The Freundlich isotherm (*R*^2^ = 0.95 M and *R*^2^ = 0.96 JF) and pseudo-first-order kinetics (*R*^2^ = 0.97 M and *R*^2^ = 0.97 JF) provided the best fit to the experimental data. Similar values of the coefficients have been observed by other researchers (Yao et al., 2014; Idrees et al., 2018; Joshi et al., 2019). Thus, we interpreted sorption following the Freundlich and Tempkin models. The Cr heterogeneously adsorbed on the biosorbent surface.

From Table 3 (Comparative equilibrium parameters of different models and their regression coefficients), the present study gives better values than others, such as activated carbon (Yao et al., 2014), bio-char (Idrees et al., 2018), bio-char (Idrees et al., 2018), and seed (Aregawi and Mengistie, 2013). It assumes the extent of non-linearity among solution composition and adsorption of chromium metal into the adsorbent plane and displays no homogeneity regarding binding location distribution.

### Effects of Contact Time, Adsorbent Doses, pH, and Initial Chromium Concentration

The chromium removal percentage was calculated concerning initial concentration and fed to the DOE for statistical analysis. The statistical analysis predicts a quadratic model that relates dependent and independent variables (pH, adsorbent doses, and metal ions of chromium) for the mango seed catalyst as shown in the model below (10):


(10) $$\begin{array}{ccc} \%removal=+42.61+2.04\times \mathrm{pH}+0.92\times \mathrm{CD}+35.30\times \text{C} & & \\ -0.148\times {\text{pH}}^{2}-0.00316\times {\text{CD}}^{2}-7.28\times {\text{C}}^{2}+ & & \\ \;\,0.0045\times \text{pH}\times \text{CD}-0.55\times \text{pH}\times \text{C}-0.16\times & & \\ \;\,\text{CD}\times \text{C} & & \end{array}$$

The dependency of percentage removal on the variation of three parameters [pH, catalyst doses (CD), and concentration (C) of heavy metal] can be seen in the given model. As the model *F* value is 11.48, it shows that the model is significant. The model *R*^2^ value is 0.93. By using JF catalyst as an adsorbent, experiments have been designed similarly and the quadratic model is presented in Eq. 11, as shown below:


(11) $$\begin{array}{ccc} \%removal=+26.5+1.53\times \mathrm{pH}+0.85\times \mathrm{CD}+48.85\times \text{C} & & \\ \;\,-0.16\times {\text{pH}}^{2}-0.00293\times {\text{CD}}^{2}-9.37\times {\text{C}}^{2}+ & & \\ \;\,0.0023\times \text{pH}\times \text{CD}-0.0366\times \text{pH}\times \text{C}-0.35 & & \\ \;\,\times \text{CD}\times \text{C} & & \end{array}$$

This model is developed by using a JF catalyst, which shows the extent of dependency of the dependent variable (% removal) on independent parameters [pH, catalyst doses (CD), and concentration (C) of heavy metal]. The model *F*-value of 12.35 implies that the model is significant. The model regression values were approximately 0.94.

**FIGURE 6:**
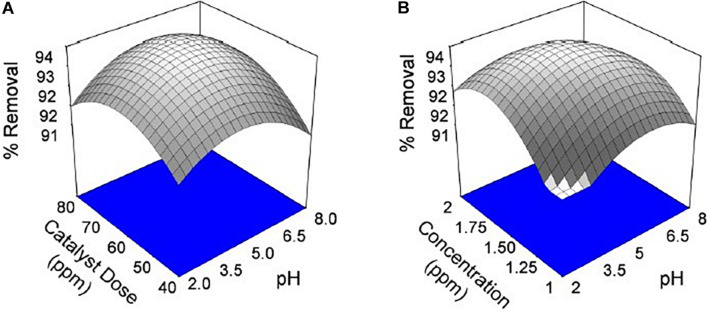
Effect of chromium by using seed bio-sorbent of Mango (M) and Jackfruit (JF): **(A)** adsorbent dose and pH and **(B)** concentration and pH of solution.

**FIGURE 7:**
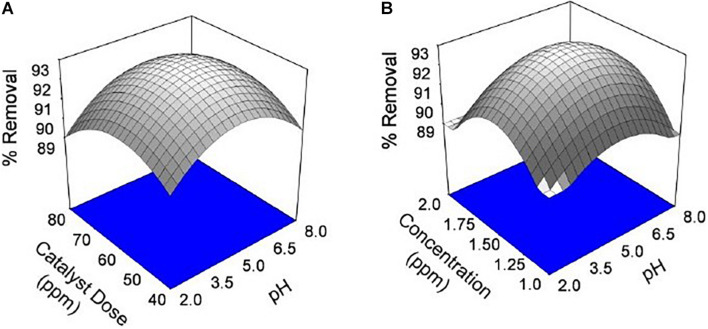
Effect of chromium by using seed bio-sorbent of Mango (M) and Jackfruit (JF): **(A)** dose and pH of solution and **(B)** concentration and pH of solution.

The dependence of percentage removal of Cr VI by the catalyst is shown in Figures 6A,B, 7A,B. The % adsorption was linearly from 15 to 80 min, and thereafter, it is approximately constant, representative of the achievement of adsorption stability. The adsorption rate was higher at the starting levels, which can be caused by the large active sites available on the bio-adsorbent plane for chromium adsorption. The sorption quickly happens and is generally measured by the diffusion procedure from the bulk to the surface. Through the increase of time, the active sites are being drenched with more chromium ions. It gives a decline in the adsorption speed until the adsorption stability is achieved.

**FIGURE 8:**
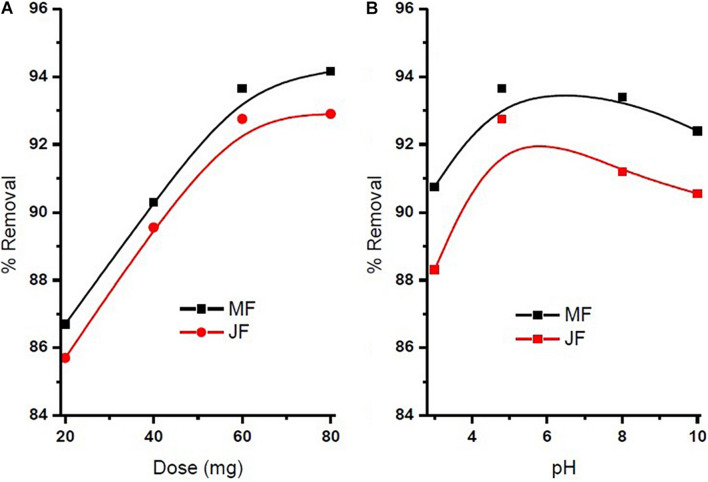
**(A)** Influence of seeds bio sorbent dosage on chromium % removal and **(B)** influence of solution pH on chromium % removal (contact time = 80 min, pH = 4.8, initial concentration = 2.5 mg⋅L^−1^, agitation speed = 400 rpm, room temperature).

**FIGURE 9:**
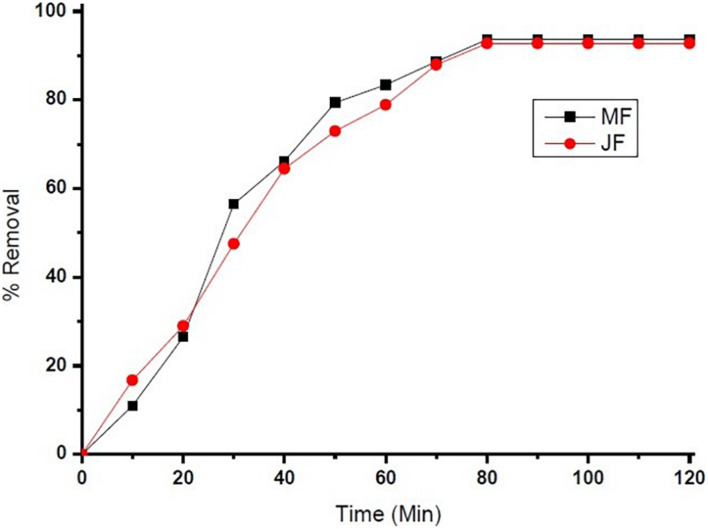
chromium % removal on Mango (MF) and Jack fruit (JF) kernel biomass (contact time = 80 min, pH = 4.8, initial concentration = 2.5 mg. L^–1^, agitation speed = 400 rpm, room temperature).

The pH of the solution is an important parameter that affects the adsorption process by affecting the sorption behaviour of adsorbate and adsorbent. The ionization of valuable groups on the bio-adsorbent plane depends on the pH (Pal et al., 2018; Prasad et al., 2020). The consequence of pH on the % of adsorption of chromium by M and JF bio-adsorbent at different pH significance is presented in Figure 8B. It was shown that a spiky increase in chromium removal happens when the pH value varies from 3 to 4.8. Further increase in pH reduced adsorption of chromium ions. The highest adsorption was at pH 4.8. The value of pH is 4.8, found to be an optimal solution from DOE. Figures 6A,B, 7A,B also show that the removal efficiency continuously increases as the pH increases up to 4.8. Therefore, all the experiments were further performed at pH 4.8. This adsorption behavior of chromium at various pH values can be attributed to the survival of chromium in various oxyanion appearances. The pH of synthetic wastewater is an important constraint in monitoring the chromium adsorption procedure (Issa et al., 2010). The variation of bio-adsorbent doses (20, 40, 60, and 80 mg) on the adsorption of chromium by the composite is presented in Figures 6–8. The % of chromium adsorption improved with an increase in bio-adsorbent dose because of more active sites and superior ease of use of plane binding sites to the biomass. Additionally, the highest chromium removal (∼94% for M and 92% JF) was recorded at the 60-mg dose. Further changes in dose did not affect percentage adsorption. Muibat et al. (2020) employed cobalt ferrite-supported activated carbon and showed chromium removal efficiency that attained equilibrium in 80 min at pH around 5. The maximum adsorption capacity of the material was approximately 23.6 mg/g following Freundlich isotherm model fitting with an *R*^2^ of 0.94 (Muibat et al., 2020). The requirement of chromium reduction on the bio-adsorbent dose was studied at pH 4.8 in 50 ml of chromium synthetic wastewater at room temperature for more than 1 h in the range of 0.05–0.215 mg/L adsorbent dosage. The % removal with contact time increases with increased time until the equilibrium adsorption is approximately 80 min, pH 4.8, and other constraints such as initial concentration = 2.5 mg/L and agitation speed = 400 rpm at room temperature, as shown in Figure 9. Padmavathy et al. (2016) have investigated the magnetite nanoparticles for chromium removal and achieved around 72% using the Langmuir isotherm model at a contact time of approximately 120 min. Agegnehu et al. (2018) studied the low-cost adsorbent vesicular basalt volcanic rock for chromium removal and showed a maximum adsorption capacity of 79.20 mg/kg at lower acidic pH around 2 and also showed pseudo-second-order kinetics.

Table 4 shows a summary of the present study and others in terms of adsorption capacity, pH ranges, contact time, and percentage removal. From all results, we can see the better percentage removal from all bio-adsorbents with less contact time and maximum adsorption capacity. These bio-adsorbents are amaltas seeds (Giri et al., 2021), ion imprinted polymer (Samah et al., 2020), rice husk (Agrafioti et al., 2014), Jamun seeds (Giri et al., 2021), and mulberry wood (Zama et al., 2017).

**TABLE 4:** Comparison between present study and reported adsorbents materials for the adsorption of chromium ions.

**Bio-adsorbents**	**Adsorption capacity (mg/g)**	**pH range**	**Adsorption time (h)**	**% Removal**	**References**
Rice husk	2.59 (μg/g)	4–9	4	95	Agrafioti et al., 2014
Mulberry wood	5	2–11	6	<42	Zama et al., 2017
Amaltash seeds	1.42	5–11	2	91	Giri et al., 2021
Jamun seeds	1.45	5–11	2	93	Giri et al., 2021
Ion imprinted polymer	0.0679 mmol/g	1–11	4	90	Samah et al., 2020
Mango seeds (MF)	5.21	5–11	2	94	Present study
Jackfruit seeds (JF)	2.23	5–11	2	92	Present study

## Conclusion

Presently prepared seed kernel bio-sorbents of mango (M) and jackfruit (JF) are well suited for chromium removal from the aqueous solution. The batch experiment at room temperature shows bio-adsorbent Cr VI removal of 94% (M) and 92% (JF) in 80 min at pH 4.8 with an equilibrium adsorption capacity of 5.21 and 2.23 g/mg, respectively. The chromium adsorption behavior of the seed’s bio-sorbent was well interpreted in terms of the Freundlich model, with a maximum adsorption capacity of 517.24 and 207.6 g/mg in 80 min for M and JF, respectively, whereas the pseudo-first-order model and Freundlich model were able to describe the kinetic curves. The work may utilize industrially to 60% recovery Cr from wastewater.

## Data Availability Statement

The raw data supporting the conclusions of this article will be made available by the authors, without undue reservation.

## Author Contributions

DP conducted all experiments, processed experimental data, and prepared the manuscript. DG, MS, NS, AH, and EA_A helped to finalize kinetic study in the manuscript. All authors contributed to the article and approved the submitted version.

## Conflict of Interest

The authors declare that the research was conducted in the absence of any commercial or financial relationships that could be construed as a potential conflict of interest. The handling editor declared a shared affiliation with the authors AH and EA_A at the time of the review.

## Publisher’s Note

All claims expressed in this article are solely those of the authors and do not necessarily represent those of their affiliated organizations, or those of the publisher, the editors and the reviewers. Any product that may be evaluated in this article, or claim that may be made by its manufacturer, is not guaranteed or endorsed by the publisher.
